# Snakebite Envenomations In The Brazilian Amazon: A Little Less Neglected

**DOI:** 10.1590/0037-8682-0462-2025

**Published:** 2026-06-22

**Authors:** Jacqueline Sachett, Fan Hui Wen, Allyson Guimarães da Costa, Felipe Murta, Erica Silva Carvalho, Felipe Queiroz Araújo, Vinícius Azevedo Machado, Alexandre Vilhena da Silva, Pedro Ferreira Bisneto, Fernando Val, Luiz Carlos de Lima Ferreira, Elder Figueira, Marco Aurélio Sartim, Wuelton Monteiro

**Affiliations:** 1 Universidade do Estado do Amazonas, Escola Superior de Ciências da Saúde, Manaus, AM, Brazil.; 2 Fundação de Medicina Tropical Dr. Heitor Vieira Dourado, Departamento de Ensino e Investigação, Manaus, AM, Brazil.; 3 Instituto Butantan, Centro Bioindustrial, São Paulo, SP, Brazil.; 4 Universidade Federal do Amazonas, Instituto de Ciências Biológicas, Manaus, AM, Brazil.; 5 Fundação de Vigilância em Saúde Dra. Rosemary Costa Pinto, Departamento de Vigilância Ambiental, Manaus, AM, Brazil.; 6 Universidade Federal do Amazonas, Faculdade de Ciências Farmacêuticas, Manaus, AM, Brazil.; 7 Duke University, Duke Global Health Institute, Durham, NC, USA.

**Keywords:** Snakebites, Epidemiology, Neglected diseases, Amazonia, Research agenda

## Abstract

Snakebite envenomations have a disproportionate burden in the Brazilian Amazon compared to other regions of Brazil. Studies conducted in the state of Amazonas show that *Bothrops atrox* snakebites are the most common, occurring in every municipality of the state. All the 62 municipalities in the state have at least one urban health unit that offers antivenom treatment. The most affected populations in terms of incidence and fatality rate are riverine communities and indigenous people, who have limited access to antivenom treatment. A significant proportion of cases are not reported to the official surveillance system, and many deaths occur due to lack of medical care. In the last decade, important advances have been made in research into snakebites in the state of Amazonas, in a collaborative network led by the Fundação de Medicina Tropical Dr. Heitor Vieira Dourado, a referral institution for patient care, teaching and research in tropical medicine. Findings on the burden of disease in vulnerable populations; clinical, therapeutic and pathophysiological aspects; long-term disabilities; barriers to antivenom treatment; and cultural aspects of snakebites, are presented in this paper. We highlight the findings of the SAVING Program, which aimed to implement a culturally tailored antivenom decentralization program in indigenous community-health centers. We conclude by presenting a priority research agenda for snakebites for the coming years in the Amazon region.

## TEMPORAL EVOLUTION AND CURRENT SITUATION OF SNAKEBITE ENVENOMATIONS IN THE STATE OF AMAZONAS

The first referral hospital for infectious and parasitic diseases in the state of Amazonas was the Instituto de Medicina Tropical de Manaus, which was founded in 1974 and is currently known as Fundação de Medicina Tropical Doutor Heitor Vieira Dourado (FMT-HVD). This hospital was also the referral center for the treatment of envenomations caused by animals. At that time, the antivenom (AV) distribution was not centralized by the Brazilian Ministry of Health (BMoH), and notification of snakebites was not mandatory. 

In 1986, the BMoH established the National Program for the Control of Snakebites, taking full responsibility for acquiring and distributing the antivenoms produced by the State-run manufacturers. The program also standardized protocols for diagnosis and treatment, developed educational activities and performed studies of snakes of medical importance^1^. FMT-HVD created the Center for Venomous Animals, responsible for distributing antivenoms and to systematize epidemiological surveillance for snakebites at the state level. Scientific studies have been developed in snake biology and ecology, and the center now maintains a herpetological collection, in addition to live specimens for collecting venom for toxicological studies (Collection of Animals of Medical Interest - ANIME, registered in the Brazilian Biodiversity Information System, in 2022).

**FIGURE 1:**
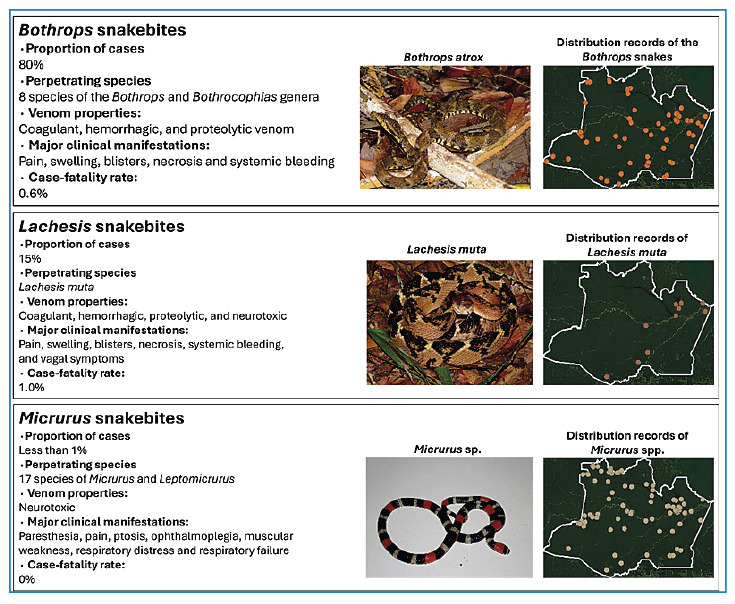
Venomous snakes of epidemiological importance in the state of Amazonas. Number of species refers only to the state of Amazonas, according to Nogueira et al.^3^ proportion of snakebites by species and case-fatality rates are based on SINAN (2025)^4^. Photo credits: *Bothrops atrox*, *Crotalus durissus* and *Micrurus* sp.; *Lachesis muta*.

Until 2004, FMT-HVD was responsible for the surveillance of snakebites and the distribution of antivenom, when the Fundação de Vigilância em Saúde do Amazonas Dra. Rosemary Costa Pinto (FVS-RCP) was created and assumed the surveillance activities within the state of Amazonas, including snakebites. The FVS-RCP is also responsible for the distribution of immunobiological products. The FMT-HVD remains the referral center for the treatment of snakebites and implements training courses for healthcare professionals. Since 2006, notifications of snakebites have been entered into an electronic database, which allows for faster analysis of snakebite envenomations (SBEs) and AV distribution in the state^2^. These data are summarized in Figure 1, combined with snake distribution^3^. Each municipality is responsible for the storage of AVs and their transport to the health units. The amount of AV destined for each municipality is established by the Zoonoses Department of FVS-RCP, based on the SINAN reports^4^. 

**FIGURE 2:**
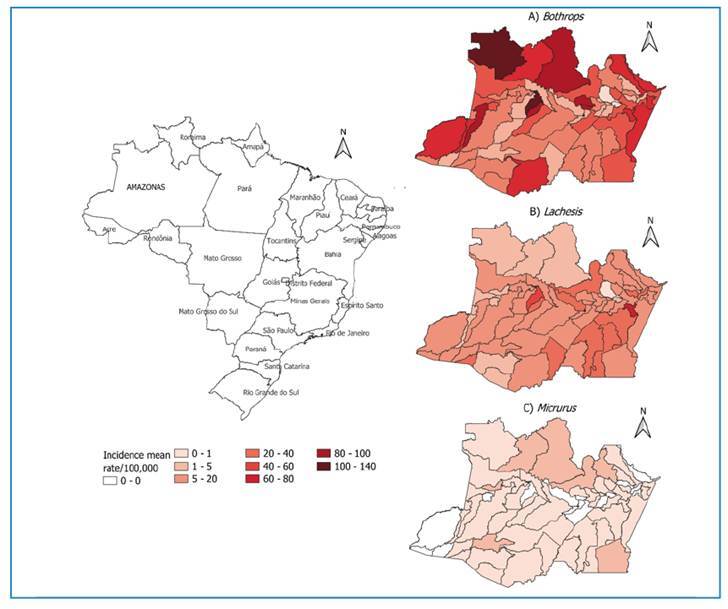
Geographic distribution of snakebites caused by *Bothrops*, *Lachesis* and *Micrurus* in the state do Amazonas, by municipality (mean annual incidence from 2007 to 2024.

*Bothrops* and *Lachesis* snakebites are reported in all 62 municipalities, with a hotspot in São Gabriel da Cachoeira (on the Colombian border), with more than 100 cases per 100,000 inhabitants/year. *Micrurus* snakebites are reported in low incidences in 47 municipalities (Figure 2).

**FIGURE 3:**
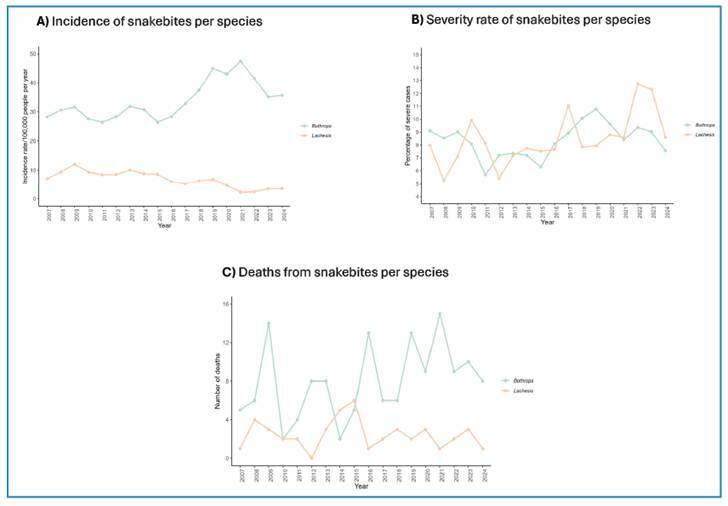
Burden of *Bothrops* and *Lachesis* snakebites reported in the state of Amazonas, from 2007 to 2024.

Figure 3 shows SBE data for the state of Amazonas, from 2007 to 2024. An increasing incidence of *Bothrops* envenomations has been observed, while *Lachesis* envenomations have decreased. This may be explained by the improvement of the surveillance system, as these two types of snakebites may be clinically similar, leading to previous overreporting of *Lachesis* envenomations^5^. The severity rate (~9% on average) and the number of deaths fluctuated throughout the period.

## MAPPING OF RESOURCES FOR TREATMENT AND CONTROL OF SNAKEBITES

All 62 municipalities in Amazonas offer AV treatment in 102 healthcare units (HCUs). The majority are hospitals located in urban areas (66; 64.7%) and 10 (9.8%) are military health units (on the borders of Peru and Colombia). More recently, 26 HCUs in indigenous territories (25.5%) started to offer AV treatment. Antivenom treatment outside of hospitals is exceptional and was only initiated recently after a successful pilot project conducted by our group^6^. 

Patients and families often access to HCUs for snakebite treatment independently, either by car or boat^7^. In Manaus and its surrounding region, there are ambulances from the Mobile Emergency Care Service (SAMU), while in riverine and indigenous communities, transportation is usually done by boats. SAMU river transport is restricted to the area around Manaus, and in a limited number of indigenous districts, there are ambulance service motorboats operated by the SAMU for Indigenous Health. There are also some HCUs in areas so remote that transport is only possible by air^2^.

Severe SBE patients are usually transferred to Manaus. In 2021, a project to expand ICU beds was initiated to the municipalities of Humaitá, Tefé, Tabatinga and Parintins^8^.

## EVOLUTION OF SCIENTIFIC KNOWLEDGE AND PROFESSIONAL TRAINING FOR THE TREATMENT AND CONTROL OF SNAKEBITES

In the first decades of the FMT-HVD, pioneer contributions were based on the biology of the snakes, and the biochemical composition and biological effects of the venom, and the description of the clinical aspects of *Bothrops atrox* envenomations^9^ ^-^ ^11^.

In August 2013, FMT-HVD performed a SWOT analysis, which highlighted the need to explore clinical studies into snakebites as a new avenue of research at FMT-HVD. The need to develop skills within the Postgraduate Program in Tropical Medicine, maintained by FMT-HVD in partnership with the Universidade do Estado do Amazonas (PPGMT), was also identified. A systematic review showed only two articles on snakebites in the state of Amazonas had been published^5^. In September of the same year, a workshop was held in Manaus, with the objective of identifying research bottlenecks and enhancing collaborative and multicenter studies on snakebites and scorpion stings. The research network Snakebites and Scorpion Stings Network in the Amazon (*Rede de Ofidismo e Escorpionismo da Amazônia*-ROdA) emerged from this meeting^12^.

In 2014, the PPGMT included Envenomations by Animals as one of its lines of research, and students started developing projects on various aspects of snakebite envenomations (SBEs). In 2015, a National Academic Cooperation Program (PROCAD) supported by the Coordenação para o Aperfeiçoamento do Pessoal de Nível Superior (CAPES) was approved, which included the PPGMT, and the postgraduate programs of the Instituto Butantan, the Universidade Federal do Tocantins, and the Universidade Federal do Acre. PROCAD strengthened postgraduate programs in the Amazon region, with emphasis on translational aspects of SBEs. 

In 2016, the Clinical Research Center for Animal Envenomation (CEPCLAM) was created at FMT-HVD, making this an official research group in the Directorate of Research Groups of the National Council for Scientific and Technological Development (CNPq). By September 2025, the senior CEPCLAM members had supervised 12 PhDs and 40 MSc degrees at PPGMT and another five postgraduate programs. Currently, 14 PhD candidates and 14 master’s students are under supervision. Two theses received national honorable mentions. The group also works on training health professionals in residency programs and offers an elective course on venomous animals for undergraduate health students at the Amazonas State University.

## MAJOR SCIENTIFIC ACHIEVEMENTS

### Burden of snakebites and risk groups

The annual incidence rate of SBEs in Amazonas is 50 cases/100,000 inhabitants^13^. The highest rates occur in São Gabriel da Cachoeira, Uarini/Alvarães and Novo Airão, with more than 150 cases/100,000 inhabitants^13^. As expected, middle aged males are more susceptible to SBEs^14^ ^-^ ^17^. An incidence rate of 334/100,000 was reported in indigenous villages, 7.5 times higher than in the non-indigenous population^18^, with an estimated underreporting rate of 23% in the indigenous health districts (18). In the Upper Solimões River and Upper Negro River, the underreporting rates were 5% and 30%, respectively^17^. In riverine communities, it was 30% along the Solimões and Purus Rivers, and 70% in communities along the Juruá River^16^. The incidence of *Bothrops*envenomations also correlated with the preserved original vegetation cover, heaviest rainfall and higher relative humidity of the air^19^. A seasonal variation is reported, with predominance in the rainy months^13^.

Case-fatality rate for SBEs in Amazonas is 0.6%, 0.7% due to*Bothrops*and 0.6% due to*Lachesis*envenomations. Age ≥65 years and time to medical assistance >6 hours are associated with the risk of death^13^ ^,^ ^20^. The case-fatality rate for *Bothrops* envenomations is about three times higher in indigenous villagers (1.4%) compared to non-indigenous populations (0.5%)^18^. A study combining data from SINAN with the Mortality Information System showed that a distance from Manaus >300 km, age ≥61 years, being indigenous, and a lack of antivenom administration were independently associated with the case-fatality rate. A total of 22% of SBE patients died with no medical assistance^21^. Many snakebite deaths occurred in remote areas, without medical assistance, which underestimates the case-fatality rate^16^ ^,^ ^17^.

The economic burden of snakebite in the Brazilian Amazon was estimated to be almost US$ 8 million in 2015, mostly associated to health system costs, premature death, and loss of productivity due to absence from work^22^.

### Clinical aspects of snakebites and complications

Our results show that, although *B. atrox*snakebites may result in ‘dry bites’^23^, patients mostly present local pain and edema starting minutes after the bite^24^. Bleeding from the fang marks is observed in 50% of cases, ecchymosis around fang punctures in 20%^25^, and enlargement of the lymph nodes of the region and bruising can also be observed in 33% of the cases^26^. Local blistering and tissue necrosis may appear within the first 24 hours^27^ ^,^ ^28^. In Manaus, secondary bacterial infection was observed in 40% of the*Bothrops*envenomations, caused mostly by *Morganella morganii* ^29^ ^,^ ^30^ *.* Tissue necrosis is more frequent when a tourniquet is used, and is associated with delayed treatment^31^. Compartment syndrome was an uncommon complication^22^ ^,^ ^32^ ^,^ ^33^, being more frequent in younger patients, those bitten in the lower limbs, those who live in rural areas, and time until care was received exceeding 6 hours^34^. Envenomations inflicted by adult snakes cause more severe local effects, whereas venom-induced consumption coagulopathy (VICC) is more frequent in SBEs caused by juveniles^35^ ^,^ ^36^.

Systemic manifestations of *Bothrops* envenomations result mainly from VICC^25^. We observed unclottable blood in 54% of the patients, systemic bleeding in 14% and thrombocytopenia in 10% upon hospital admission; gingivorrhagia, macrohematuria and ecchymosis were the most frequent types of systemic bleeding^37^. Unclottable blood and thrombocytopenia on admission were associated with systemic bleeding. Low levels of fibrinogen and alpha 2-antiplasmin, and high levels of fibrin/fibrinogen degradation product (FDP) and D-dimers were detected^25^. Platelets decreased in the first 24-48 hours after hospital admission^25^ ^,^ ^26^. CNS bleeding occurred even with clottable blood and normal platelet counts^38^ ^-^ ^40^. Subclinical myocardial injury correlated with VICC^41^. Ischemic stroke^42^ and acute mesenteric ischemia were also reported in severe cases^43^. Acute kidney injury is observed in 13% of the patients in Manaus^44^. 

Severe complications of *B. atrox* envenomations in pregnant women have been described, such as placental abruption and premature birth^45^, and an increased risk of fetal and neonatal deaths^46^. 

Confirmed cases of *Lachesis* envenomations are rare and can be misdiagnosed as *B. atrox* envenomations, as clinical manifestations are similar^47^. Similarly, many *B. atrox* SBE are misdiagnosed as *Lachesis* bites, as the two snakes share the same common name in several regions of the Amazon^48^. Envenomations by coral snakes are rare, mostly mild cases presenting local pain, edema and paresthesia^49^. 

Non-venomous snakes are commonly observed in the Brazilian Amazon, and their bites can cause pain, bleeding from the fang marks, bruising and mild edema^50^ ^-^ ^52^. The color of some non-venomous species can generate incorrect *Bothrops* snakebite diagnoses.

### Long-term disabilities from snakebites

The complications from snakebites may cause severe long-term disabilities. Scarring, muscular atrophy and amputation that affects mobility are potential outcomes after compartment syndrome and/or extensive tissue necrosis^32^ ^,^ ^33^. Children <12 years present a higher probability of developing several local complications that evolve to amputations^20^. Long-term disabilities after cerebral strokes following a *B. atrox* envenomation were also reported^42^ ^,^ ^53^. In Manaus,*Bothrops*snakebites frequently cause chronic pain, loss of mobility and impaired sensory abilities, resulting in reduced quality of life of the patients^42^ ^,^ ^54^.

### Venom research and pathophysiology of snakebite envenomations

Venoms of*B. atrox* captured in different parts of the Brazilian Amazon show a differential distribution of protein isoforms, which results in functional distinctiveness and ability to occupy different habitats^55^. The major components of the venom are snake venom metalloproteinases (SVMPs), C-Type lectin-like toxins (CTL), snake venom serine proteinases (SVSPs) and phospholipases A_2_ (PLA_2_)^56^ ^,^ ^57^. We found correlations in the *B. atrox*venom proteome of a snake brought to the hospital in Manaus by a patient with clinical signs of envenomation^57^: i) SVMP group correlated with bleeding disorders and edema; and ii) some isoforms of SVMPs, CTL and SVSP expression levels correlated with bleeding disorders, edema, ecchymosis and blister formation. Transcriptomic and proteomic profiling of the venoms of *Bothrocophias hyoprora*and different *Bothrops* species collected in the Amazon rainforest indicated the same toxin groups that are characteristic of bothropoids, with qualitative and quantitative variability^56^. 

Skin histopathology revealed the occurrence of hemorrhagic intraepidermal blisters and severe necrosis in the epidermis; and hemorrhage, inflammatory infiltrate, edema, vascular damage, necrosis, abscess and signs of tissue repair in the dermis and hypodermis^28^. Proteomics of the blister fluids revealed a rich source of damage-associated molecular patterns (DAMPs), immunomodulators and matrix metalloproteinase-9 (MMP-9), suggesting that the mechanisms by which blisters are formed includes the toxins very early in envenomation and continue even after antivenom treatment^27^. Patients with severe tissue damage have a more intense local response polarized profile towards Th1 response, with high serum levels of CCL-2 and CXCL-10^58^. Higher levels of circulating IL-2, IL-10, IL-6, TNF, INF-γ and CXCL-10 were observed in patients with secondary bacterial infection, with marked correlations between these mediators and IL-4 and IL-17^59^.

Unclottable blood (58%) and thrombocytopenia (9%) were associated with systemic bleeding^37^. These patients presented lower levels of factor V, II, fibrinogen, plasminogen and alpha 2-antiplasmin, and high levels of tissue factor and FDP compared to those without bleeding^25^. Consumption coagulopathy did not correlate with venom levels^26^. Tissue factor presented a weak correlation to factor V, II, D-dimer, plasminogen, alpha 2-antiplasmin, and a moderate correlation to fibrinogen and fibrin/fibrinogen degradation product (FDP)^25^. Patients with low fibrinogen levels have higher concentrations of CCL-5 and lower IFN-γ and several interactions of CXCL-8, CXCL-9, CCL-2, IL-6 and IFN-γ, which may be related to the inflammation-coagulation mutual relationship induced by*B. atrox*venom^60^. 

Acute kidney injury (AKI) from *B. atrox* envenomation showed a positive correlation with circulating venom concentrations, suggesting a dose-dependent participation of the venom toxins in the pathogenesis of this complication^60^. Urine proteomics shows that AKI was associated with acute phase response, endopeptidase inhibition, complement cascade, and inflammation, with notable high concentrations of serotransferrin, SERPINA-1, alpha-1B-glycoprotein and NHL repeat-containing protein 3, retinol-binding protein, beta-2-microglobulin, cystatin-C and hepcidin^60^. Elevated levels of CXCL-8 and CCL-2 molecules were associated with AKI^61^. 

We studied *Crotalus durissus ruruima* venom, and the results point to a dichotomy in the venom with repercussions on biological and medical properties^62^: PIII-class, associated with proteolytic and hemorrhagic activity, predominant in the Type I venoms; crotoxin A and B chains are prevalent in Type II venoms and are related to elevated phospholipase A2 activity, causing myotoxicity and increased lethality in mice.

### Clinical research on snakebite treatment

Although Amazonian *Bothrops* venoms are not included in the pool used to immunize horses in the AVs produced by the Brazilian manufacturers, the available antivenoms are able to neutralize venom components and impair the lethality in mice^56^. Clinical follow-up of patients in Manaus indicates that the AVs are effective in reversing venom-induced coagulopathy^26^, with no pyrogenic reactions and a decreasing trend in early allergic reactions: i) 19.8% from 2004-2007^63^; ii) 11.8% from 2014 to 2016^64^; and 5.9% in 2023-2024^65^. These studies show mostly mild reactions, such as urticaria and pruritus^66^.

Clinical trials performed in Manaus with *Bothrops* envenomation patients indicated that preemptive amoxicillin clavulanate was not effective in preventing secondary infections^29^. Another trial demonstrated the efficacy of low-level laser therapy in reducing pain, local inflammatory processes and muscle damage^67^. Adjunctive heat therapy had no efficacy in treating the local effects of*B. atrox*envenomations, though cold therapy reduced pain^68^.

### Barriers to AV treatment

Geographic barriers and economic restrictions prevent patients in remote areas from seeking medical care after an SBE^7^. Few families have their own means of transport, most of them cannot keep their vehicles fueled, or afford private transport to the hospital. Public ambulances are mostly unavailable, and transport is primarily by river^16^ ^,^ ^17^. As a consequence, many villagers are treated using only traditional medicines and seek medical care only when the SBE becomes life threatening^7^ ^,^ ^17^. For indigenous populations, leaving their territories and staying away from their families for a long time in accommodation that is not suited to them, the quality of the care provided and their inability to comply with dietary restrictions required by the patient's condition represent sociocultural barriers^17^.

### Implementation of a culturally tailored AV decentralization program

As antivenom treatment in indigenous health units is a long-standing demand^18^, a program to decentralize antivenom treatment and install it in indigenous territories, through an uncontrolled quasi-experimental study, named the SAVING Program (‘Snakebite AntiVenom Immunoglobulins Need to be Guaranteed’), was designed to be trialed at fourteen SIHD health centers in seven indigenous territories in the state of Amazonas^6^. The SAVING program was based upon BMoH snakebite treatment guidelines and the National Policy for Healthcare for Indigenous Peoples, in which all patients receive antivenom in their own indigenous territory, and only severe cases would be transferred to the hospital. We developed and validated a minimum-requirements checklist for SBE treatment^69^ to assess the capacity of indigenous health centers to provide AV treatment^70^. We also validated a culturally tailored clinical-practice guideline to train health professionals^71^. Our proposed solution has shown to be cost-effective^72^ and acceptable amongst managers, health professionals, indigenous healers and victims of snakebites^73^ ^-^ ^75^. Telehealth tools have helped in the healthcare process^76^. 

### Anthropological aspects of snakebites

Surveys with riverine and indigenous people identified several medicines of plant and animal origin, as well as mythical-religious rituals used to treat snakebites^16^ ^,^ ^17^. Our group is also working to build explanatory models of the indigenous healthcare domain for snakebite patients. In the Upper Solimoes, snakes are intentional beings and share a cultural and psychological background with humans from indigenous caregivers’ perspective. Snakebites have a natural or a supernatural cause, the latter being more difficult to prevent and treat. Severe or lethal snakebites are understood as having been triggered by sorcery. Treatment includes immediate self-care using parts of the snake, rituals and medicines derived from plants and animals, transfer to a health unit to receive antivenom, and care in the village after hospital discharge, also using traditional medicines. All these phases are intertwined with dietary taboos and behavioral interdictions (avoiding contact with menstruating or pregnant women) to prevent complications and death^75^. 

## PRIORITY RESEARCH AGENDA OFOR SNAKEBITES FOR THE COMING YEARS

Despite important efforts being carried out during the past decades to understand and face the problem of snakebites in the state of Amazonas, obstacles remain if we are to halve snakebite deaths and disabilities. There is an urgent need for researchers, policy makers and funders to align their research with public health priorities. Here we present a priority research agenda on snakebites considering the needs of the Amazon.

### Interventions to increase access to antivenom treatment

- Assess effectiveness, acceptability, and other feasibility outcomes using the SAVING Program.
- Investigate alternatives to reduce treatment delays, including AV treatment where the bite occurred or during transport in ambulances.
- Develop training materials and validate user-friendly virtual packages for healthcare professionals working in remote areas.
- Develop a validated telehealth program, connecting professionals working in CHCs with specialists.
- Develop culturally tailored preventive strategies for indigenous and riverine communities, integrating community-collected data with the health system.

### Climate changes, environment degradation and snakebite research

- Investigate SBEs in the One Health perspective, involving biotic and abiotic components of the Amazonian biodiversity.
- Estimate the effect of seasonal fluctuations of temperature and river levels, climate change and loss of biodiversity on the snake fauna and snakebite burden.
- Consolidate a repository of snake ecology and diversity, SBE incidence, and climate data and build predictive tools to assess the impact of climate change on SBEs.
- Evaluate and enhance the resilience of the health system in responding to changes in SBE epidemiology during extreme events.

### Pathophysiology and clinical aspects of snakebites

- Explore the cellular, molecular and immune mechanisms involved in local manifestations.
- Estimate the burden and explore pathophysiology of neurological damage associated with long-term neuropsychological disabilities.
- Identify predictors and biomarkers for long-term disabilities.
- Develop and validate new POC diagnostic tools to detect VICC.
- Build a multicenter biorepository of clinical samples in the Amazon to support hypothesis-driven projects on pathogenesis, diagnostics and prognostics.

### Therapeutical aspects and rehabilitation

- Develop and validate high-throughput screening methods to identify new small-molecule toxin inhibitors.
- Develop models for antivenom pharmacokinetics and toxicokinetic studies of *B. atrox* venom toxins in patients.
- Perform clinical trials (CTs) on toxin inhibitors, for pre-hospital treatment of snakebites.
- Perform CTs on antibiotics to treat secondary bacterial infections resulting from snakebites.
- Perform CTs on rehabilitative techniques for long-term disabilities.
- Establish a continuous sentinel-based observatory of the safety of antivenoms, including early and late adverse reactions.

### Population engagement, education and cultural aspects

- Empower communities to respond to snakebite events, promoting equity in snake-human-environment relationships.
- Codevelop bilingual multimedia resources, interactive learning methods, and technology platforms for indigenous groups.
- Build explanatory models for snakebites from the indigenous perspective.

## FINAL REMARKS

Important advances have been made in research on SBEs in the state of Amazonas in the last decade. These studies have led to changes in the BMoH's clinical protocols and in the country's antivenom treatment policy. A priority research agenda for snakebites should be carried out via multidisciplinary studies, engaging researchers, policy makers, health professionals and communities. Strengthening regional networks, fostering multicenter collaboration, and aligning scientific priorities with public health needs are essential to achieve the WHO goal of halving deaths and disabilities from snakebites by 2030 in the Amazon.

## Data Availability

Research data is available in the body of the article.
